# Strains of bacterial species induce a greatly varied acute adaptive immune response: The contribution of the accessory genome

**DOI:** 10.1371/journal.ppat.1006726

**Published:** 2018-01-11

**Authors:** Uri Sela, Chad W. Euler, Joel Correa da Rosa, Vincent A. Fischetti

**Affiliations:** 1 Laboratory of Bacterial Pathogenesis and Immunology, The Rockefeller University, New York, New York, United States of America; 2 Department of Medical Laboratory Sciences, Hunter College, CUNY, New York, New York, United States of America; 3 Laboratory for Investigative Dermatology, The Rockefeller University, New York, New York, United States of America; Duke University Medical Center, UNITED STATES

## Abstract

A fundamental question in human susceptibility to bacterial infections is to what extent variability is a function of differences in the pathogen species or in individual humans. To focus on the pathogen species, we compared in the same individual the human adaptive T and B cell immune response to multiple strains of two major human pathogens, *Staphylococcus aureus* and *Streptococcus pyogenes*. We found wide variability in the acute adaptive immune response induced by various strains of a species, with a unique combination of activation within the two arms of the adaptive response. Further, this was also accompanied by a dramatic difference in the intensity of the specific protective T helper (Th) response. Importantly, the same immune response differences induced by the individual strains were maintained across multiple healthy human donors. A comparison of isogenic phage KO strains, demonstrated that of the pangenome, prophages were the major contributor to inter-strain immune heterogeneity, as the T cell response to the remaining “core genome” was noticeably blunted. Therefore, these findings extend and modify the notion of an adaptive response to a pathogenic bacterium, by implying that the adaptive immune response signature of a bacterial species should be defined either per strain or alternatively to the species’ ‘core genome’, common to all of its strains. Further, our results demonstrate that the acquired immune response variation is as wide among different strains within a single pathogenic species as it is among different humans, and therefore may explain in part the clinical heterogeneity observed in patients infected with the same species.

## Author summary

We address a fundamental question regarding the variability in human susceptibility to bacterial infection. Recent attention has been directed to human immune variation as the major element in infection susceptibility, however, our study focuses on the contribution of the pathogenic bacteria. To separate the contribution of bacterial versus human variability, we compared the acute adaptive immune response (both T and B cells) induced by several strains within two human disease-causing bacterial species in the same individual, and verified in 10 other humans. Our results clearly demonstrate that different strains of a species vary widely in the acute adaptive immune response they induce. Most importantly, using mutant strains, we show that the observed variability is a function of the differences found in the bacterial accessory genome, i.e., its lysogen. Previous publications generally base their results on a single strain as the representative of a species. Our findings extend and modify this notion by implying that the adaptive immune response signature to a pathogenic species should be defined either per strain or alternatively to the species’ ‘core genome, common to all of its strains. The results, while novel and unexpected, may set the stage to ultimately better predict patient disease outcome.

## Introduction

Large intra-species variability exists in bacterial genome content. The gene collection found in all members of a species is defined as the essential ‘core’ genome, while genes that are found only in some strains are termed the ‘accessory’ genome. Gene families found within a species as a whole are considered the pangenome [1, 2]. As a result, various strains of a bacterial species are not equally pathogenic due to dissimilarity in virulence factor expression, among others [3]. Strains within a species are known to express a unique combination of virulence factors and superantigens [4], many of which are carried by prophage [5, 6], constituting a portion of the strain accessory genome. Though some of these virulence factors were shown to induce a robust T cell activation [7], it is unclear whether and how various strains within a species differentially affect aspects of the adaptive immune response. To address this: (i) blood samples from healthy donors were used to rule out primary or secondary immune deficiency as a cause for heterogeneity (ii) we evaluated the adaptive immune response of proliferating cells following stimulation with heat killed bacteria [8, 9] and, (iii) the acute adaptive immune response 4 days after initial stimulation was examined, thereby simulating the acute adaptive response to a pathogen early after its encounter.

## Results/Discussion

**Fig 1:**
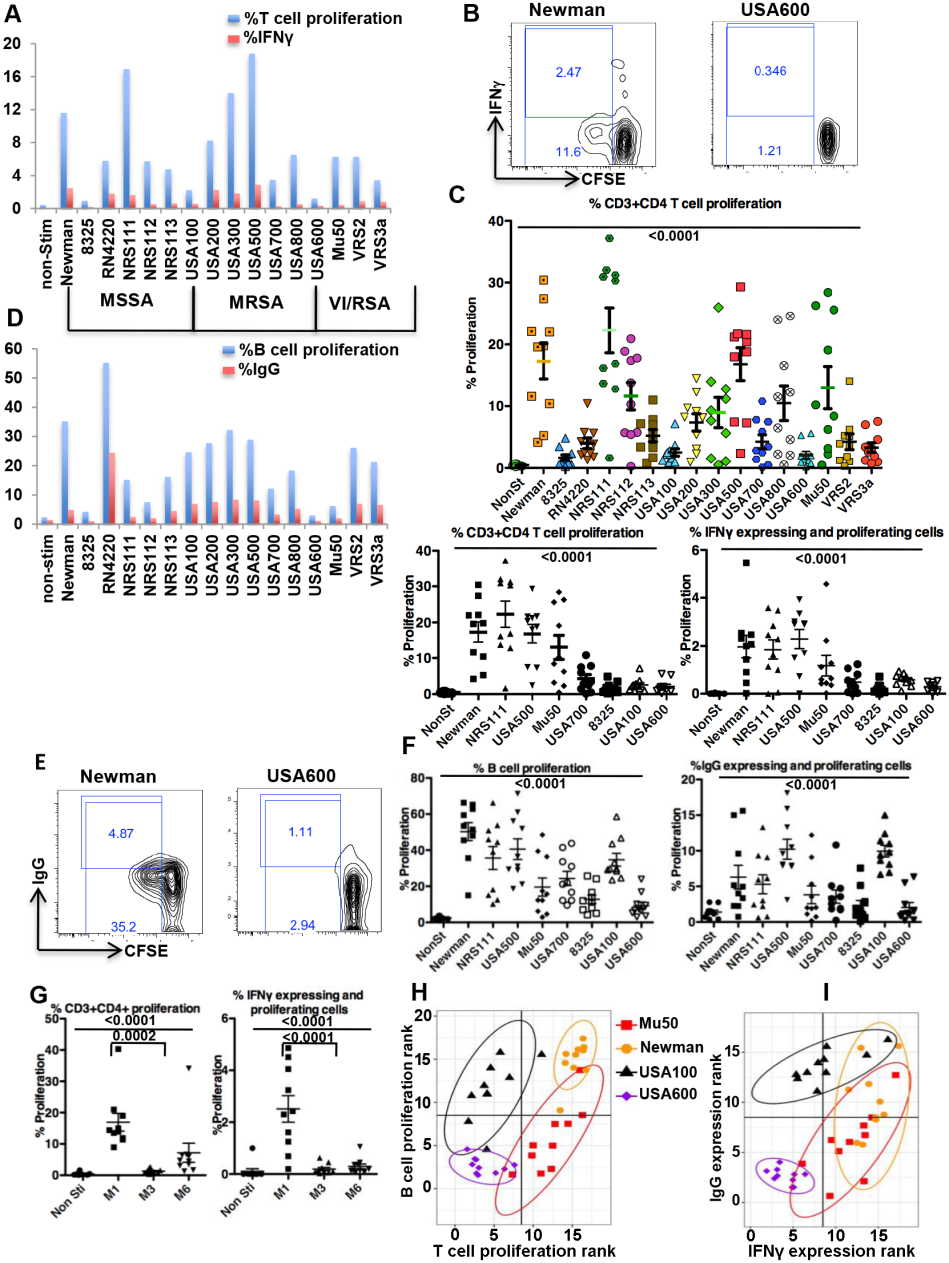
Various strains of a species induce a greatly varied acute adaptive immune response. **A**. A donor CFSE labeled PBMC were stimulated with 16 heat killed *S*. *aureus* strains and 4 days later were re-stimulated for 6 hrs after which they were stained and analyzed by FACS for percent CFSE dilution (proliferation) and IFNγ expression in proliferating cells when gated on live CD3+CD4+ cells. **B**. Example of FACS plot of 2 of 16 strains evaluated using same Figure A donor. **C**. Upper panel is the percent live CD3+CD4+ proliferation in 10 donors in response to 16 strains. The pair-wise comparison is in S1 Table. Lower panel is live CD3+CD4+ proliferation (lower left panel), and IFNγ expression in live CD3+CD4+ proliferating cells (lower right panel) in same 10 donors in response to 8 representative strains (with high, intermediate and low T cell proliferation). The results from all 16 strains for percent IFNγ expression and the complete statistical analysis are presented in the S1 Fig and S2 Table. For lower panels, Friedman statistics P<0.0001 for both, significant Dunns for pairs in Rt lower panel: for Newman vs 8325, Newman vs USA600, NRS111 vs 8325, NRS111 vs USA600, USA500 vs USA700, USA500 vs 8325, USA500 vs USA600. In left lower panel: Newman vs 8325, Newman vs USA600, NRS111 vs USA700, NRS111 vs 8325, NRS111 vs USA100, NRS111 vs USA600, USA500 vs 8325, USA500 vs USA600. **D**. Same donor as in A, but gated on live CD3-CD19+ for B cell proliferation and IgG expression in proliferating cells in response to the 16 strains. **E**. Example of FACS plot of 2 of 16 strains evaluated in Figure D donor. **F**. The percent B cell proliferation and IgG expression in live CD3-CD19+ proliferating cells in 10 donors in response to the 8 strains with prominent different values C. (the results from the 16 strains are in Supporting Information). **G**. The percent proliferation and IFNγ expression by live CD3+CD4+ proliferating cells stimulated with in to *Streptococcus pyogenes* strains in 10 donors. **H**. **and I**. The MANOVA test for the difference between bivariate means of ranks of T and B cells proliferation (H) and IFNγ vs IgG expression (I) among 16 strains, showed significant differences (p<0.00001). The post-hoc pairwise comparisons identified the differences between strains to be significant (P<0.0001), except for T and B cell proliferation (H) with Mu50 vs. USA 100 (p-value = 0.53), and IFNγ vs IgG expression (I) with Newman vs USA100 (p-value = 0.51).

We first assessed the T cell responses to 16 different heat killed *Staphylococcus aureus* (*S*. *aureus*) strains, either methicillin sensitive (MSSA), resistant (MRSA) or vancomycin resistant (VISA/VRSA). CD4 T cell proliferation and Interferon-gamma (IFNγ) expression by proliferating cells, denoting *S*. *aureus*-specific cells, demonstrated broad heterogeneity in response to the various staphylococcal strains within the same blood donor (Fig 1A and 1B). Further, results from 10 additional unrelated donors showed that the heterogeneity and relative intensity of T cell responses (proliferation, IFNγ expression) by the 16 strains is maintained across different donors (Fig 1C top for 16 strains, and bottom left for 8 representative strains with high, intermediate and low T cell proliferation, and S1 Table). Therefore, strains that were relatively weaker (e.g., USA 600, USA 100) or stronger (e.g., Newman, NRS111) inducers of T cell proliferation or IFNγ expression kept their relative intensity across all donors (Fig 1B and 1C, and S1 Fig, S2 Table). Furthermore, this T cell response heterogeneity was also seen within different strains of *Streptococcus pyogenes* (Fig 1G).

We next evaluated other adaptive immune functions e.g., B cell proliferation and IgG expression by proliferating cells, using the same donor as in Fig 1A in response to the same 16 *S*. *aureus* strains. To decrease bias between experiments, the PBMCs from the culture well that were stained for T cells (Fig 1A) were simultaneously co-stained for B cell markers and analyzed by FACS. Again, we observed wide heterogeneity in the intensity of B cell proliferation and IgG expression in response to the various strains (Fig 1D and 1E). Additionally, strains that induced relatively strong T cell responses (Fig 1A, e.g., NRS111, USA300) either maintain the same intensity of B cell responses (Fig 1D, e.g., USA300) or had a low B cell response (e.g., NRS111) and vise versa (e.g., USA600 maintained low, and USA100 increased). Moreover, as with the T cell responses, the relative intensity of the B cell response to the 16 strains was maintained across the same 10 donors (Fig 1F, S2 and S3 Figs and S3 and S4 Tables).

Comparing T and B cell response intensities to the 16 strains across donors, demonstrated a unique combination of adaptive immunity activation by different strains of the same *S*. *aureus* species (Fig 1H and 1I). Indeed, USA600, USA100, Mu50 and Newman, induce low/low, low/high, high/low and high/high combinations of T/B cell proliferation, respectively (Fig 1H and S5 Table).

The combination of the adaptive effector molecules IFNγ/IgG (Fig 1I and S6 Table) has a similar low/high and high/low induction pattern (for USA600 and Mu50, respectively), however with Newman and USA100 there is some “spread” showing higher variability in IgG expression. However, USA500 induces a high/high IFNγ/IgG expression pattern with low heterogeneity (S4 Fig and S7 Table). Despite the fact that all the strains we used carry a combination of virulence factors and at least one superantigen (the lab strain RN4220 has no superantigens, [10]), their *net* effect on the adaptive immune response is strikingly heterogeneous. Thus, these findings provide strong evidence that various strains of a species are markedly varied in the acute adaptive immune response they induce.

**Fig 2:**
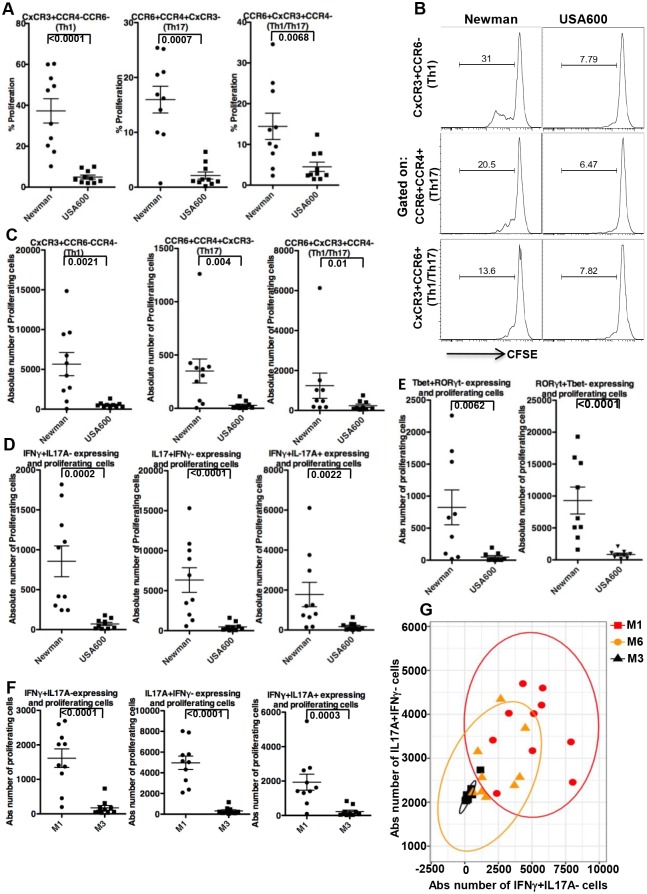
Strains of a species may differ prominently in Th1/Th17 response intensity. **A**. CFSE labeled PBMC from 10 donors were stimulated and re-stimulated as described in Fig 1A (as in Fig 1) with either Newman or USA600, stained and analyzed by FACS for percent CFSE dilution (%proliferation) in live CD3+CD4+ cells that were gated on Th subsets based on cell surface markers (CxCR3, CCR6, CCR4). **B**. Example of FACS plot of one of the donors in Figure A. **C**. Absolute number of proliferating cells in response to stimulation with either Newman or USA600, when gated on cell surface markers for Th subsets cells. **D**. Intracellular staining for IFNγ, IL17A expression in proliferating live CD3+CD4+ cells following stimulation with either Newman or USA600 as in A. **E**. As in D, but staining for transcription factor Tbet and RORγt. **F**. As in D after stimulation with *Streptococcus pyogenes* strains. **G**. The MANOVA test for difference between bivariate means of induced IFNγ+IL17A- vs IL17A+IFNγ- absolute (Abs) number of cells following stimulation with *Streptococcus pyogenes* strains among 10 donors, showed significant results (p<0.00001). The post-hoc pairwise comparisons identified the differences between strains to be significant (for M1 vs M3 and M1 vs M6, P<0.0001; for M3 vs M6 P<0.005).

To further understand the potential implication of the adaptive immune response heterogeneity to various strains of a species, we evaluated its possible relevance to immune protection. Th17 cells are crucial in preventing *S*. *aureus* infections and a low Th17 cell count is responsible for recurrent infection by this bacterium [9, 11, 12]. Further, Th1 and MRSA-specific IFNγ+ CD4 T-cell responses were shown to be essential for the control of initial and recurrent MRSA infections in HIV-infected people [13]. Therefore, we next assessed the *S*. *aureus*-specific Th response, focusing on two of the strains in Fig 1 that showed wide differences in their adaptive immune responses (Newman and USA600). Using cell surface markers (CxCR3, CCR6, CCR4) to identify the Th subsets [14], we found a dramatic difference in *S*. *aureus*-specific Th1, Th17, and Th1/Th17 subset responses to Newman compared to USA600 (Fig 2A, 2B and 2C). USA600 had at least a 10-fold lower response in both mean values of IL17A (and IL17F) as well as IFNγ-expressing cells among the CD3+CD4+ proliferating cells (Fig 2D and S5 Fig). This was accompanied by a similar trend in expression of their master transcriptional regulators in the proliferating cells, i.e., RORγt and Tbet, respectively (Fig 2E). Further, in contrast to the robust Th17 response described before in response to *S*. *aureus* [8], our findings indicate that the Th response intensity is strain dependent. Apparently, most of this effect is due to prominent differences in CD4 proliferation in response to USA 600 compared to Newman (Fig 1A, 1B and 1C), differences that take place despite the presence of superantigen genes in both strains [4, 15]. In fact, the observed 10 fold lower response with USA600 occurs despite coding for at least 7 superantigens, while Newman contains only one [4, 15]. This suggest that its not the presence or number of superantigens or specific virulence factors that contribute to the observed difference but rather the net effect of the combination of these molecules (unique to each strain) that dictate the final result on the adaptive immune response. Of note, similar variability in Th1/Th17 was also observed with other *S*. *aureus* strains (e.g., NRS111 had a 7-fold higher response in values of IL17A as well as IFNγ-expressing cells among the CD3+CD4+ proliferating cells compared to USA700). Since the effect on T cells may be affected by the donor’s previous exposure to *S*. *aureus*, we used murine CD4+T cells to identify the difference in the strain effect. We found a statistically significant difference of almost two-fold in T cell proliferation in response to Newman vs USA600 (S6 Fig).

We again verified our results with a different bacterial species. The adaptive immune response against *S*. *pyogenes* includes a robust Th1 response, and IL-17A was shown to be necessary for *S*. *pyogenes* clearance [16, 17]. Comparing *S*. *pyogenes* strains from two M serotypes, i.e., M1 and M3, demonstrates a >10-fold difference in mean strain-specific IL-17A or IFNγ CD3+CD4+ expressing cells (Fig 2F), which in large part may be attributed to effects on proliferation (Fig 1). Serotype M6 strain MGAS10394 demonstrates intermediate counts of IL17A (S7 Fig right) or IFNγ (S7 Fig left) expressing CD3+CD4+ cells, that did not differ from either serotypes M1 strain SF370 or M3 strain MGAS315, respectively. However, by performing a joint analysis of the two cytokine-expressing cells (IFNγ+IL17A-, IL17A+IFNγ-) we could identify the heterogeneity of M6 compared to the other two strains, showing significant differences in all post-hoc pairwise comparisons (M1vs M3, M1 vs M6, M3 vs M6, Fig 2G). This suggests that combined analysis of several variables may better reveal strain heterogeneity. Thus, our results from two different species strongly suggest that individual strains within a species may differ prominently in the intensity of the necessary protective Th response.

**Fig 3:**
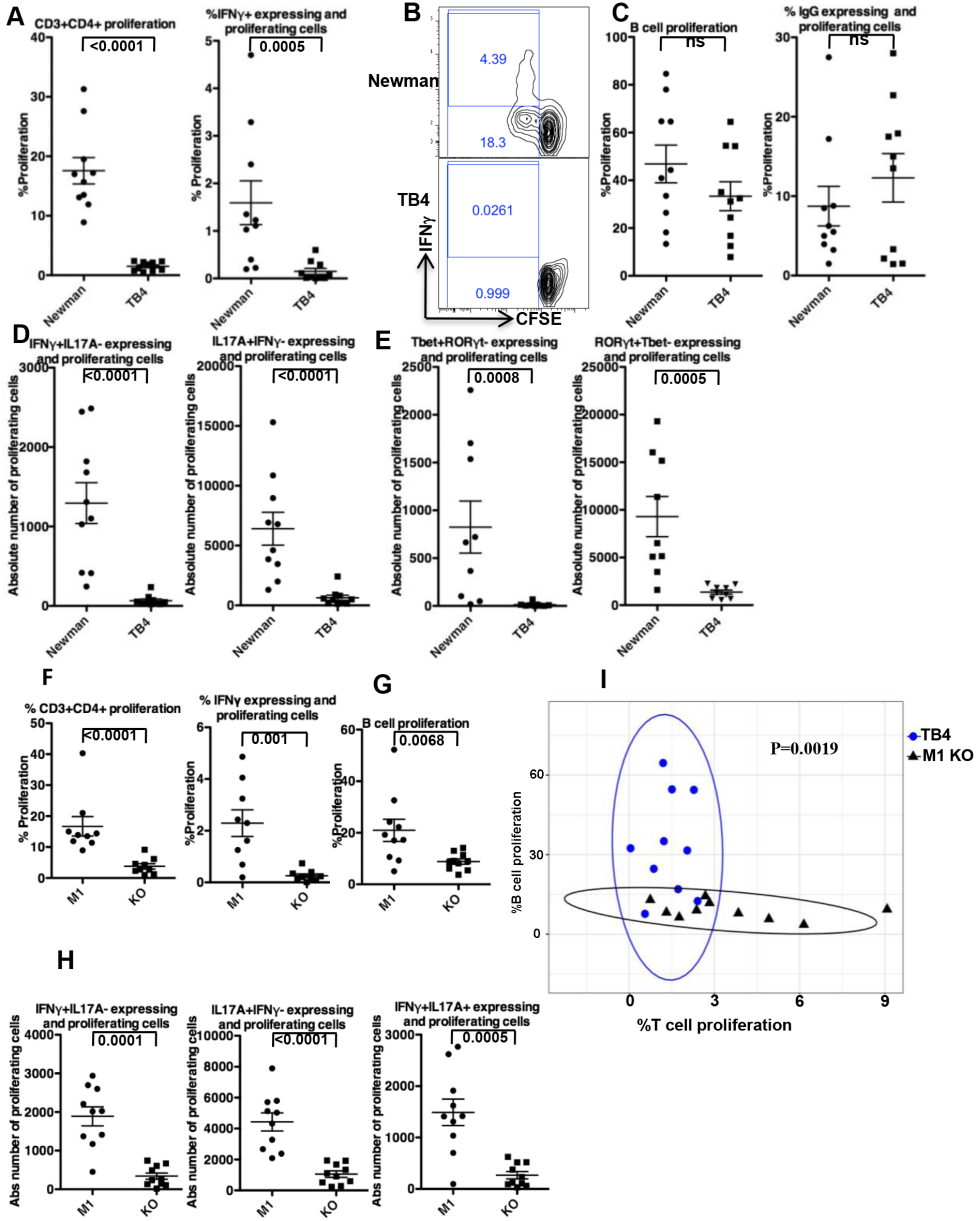
The effect of phage KO on adaptive immune response. **A. and C**. CFSE labeled PBMC from 10 donors were stimulated with strains Newman or TB4 (Newman KO of its 4 phages) as in Fig 1A, stained and analyzed by FACS for % proliferation (CFSE dilution) of live CD3+CD4+ cells (A left), % IFNγ expression by proliferating live CD3+CD4+ cells (A right), % proliferation of B cell (live CD3-CD19+, C left), or % IgG expression by live B cells (C right). **B**. Example of FACS plot of one of the donors in Figure A. **D**. Intracellular staining for IFNγ, IL17A expression in proliferating live CD3+CD4+ cells following stimulation with either Newman or TB4. **E**. As in D, but staining for transcription factor Tbet and RORγt. **F. and G**. are as in A and C left, respectively, but after stimulation with wild type *Streptococcus pyogenes* M1 strain SF370 or its complete phage KO. **H**. Intracellular staining for IFNγ, IL17A expression in proliferating live CD3+CD4+ cells following stimulation with either wild type *Streptococcus pyogenes* M1 strain SF370 or its complete phage KO (CEM1ΔΦ). **I**. The MANOVA test for difference between bivariate means of % T cell proliferation and % B cell proliferation cells following stimulation with either TB4 or *Streptococcus pyogenes* M1 (SF370) complete phage KO (CEM1ΔΦ) strains.

To determine the source of the heterogeneity in the adaptive response to the various strains of a species, we focused our attention on those parts of the bacterial genome that may account for inter-strain differences, i.e., the accessory [1, 2] genome. To this end, we compared the adaptive immune response to the Newman wild type (WT) strain of *S*. *aureus* to that of Newman strain TB4 deleted of all four of its prophages (ϕNM1-4) [18]. Regardless of the presence of plasmids, pathogenicity islands or other mobile genetic elements, prophages are a major part of the accessory genome in this species; therefore, their removal more closely reflects the “core” genome of Newman, and other strains within the *S*. *aureus* species. Newman strain TB4 induced a blunted T cell response (Fig 3A and 3B), with a striking 10-fold reduction in the means of Newman-specific Th1 and Th17 cell counts from the same donor population (Fig 3D and 3E). This suggests that antigens encoded within the accessory genome induce most of the acute Th1 and Th17 responses, considered to be necessary for protection against *S*. *aureus* infection [9, 11–13].

Using the same approach we compared the adaptive immune response to wild type *S*. *pyogenes* M1 (SF370) vs its complete phage KO (CEM1ΔΦ) [19]. Similar to *S*. *aureus* Newman and its KO, most of the T cell responses to the *S*. *pyogenes* strain M1 originate from genes found in the M1 accessory phage genome (Fig 3F and 3H). However, in contrast to Newman, a prominent portion of B cell activation is also attributed to the net effect of the accessory genome (Fig 3G). This suggests that the influence of the accessory genome is not confined to T cell responses alone, but rather, depending on the species, both arms of the adaptive immune response may be regulated by the bacterial accessory genome. Moreover, the heterogeneity of the adaptive immune response to various strains of a species may imply that there is no single immune signature (or Th) that represents a bacterial species. However, defining the adaptive immune response to a species by its “core genome”, whether practical or not, may be a more suitable alternative for comparing species (see Fig 3I).

**Fig 4:**
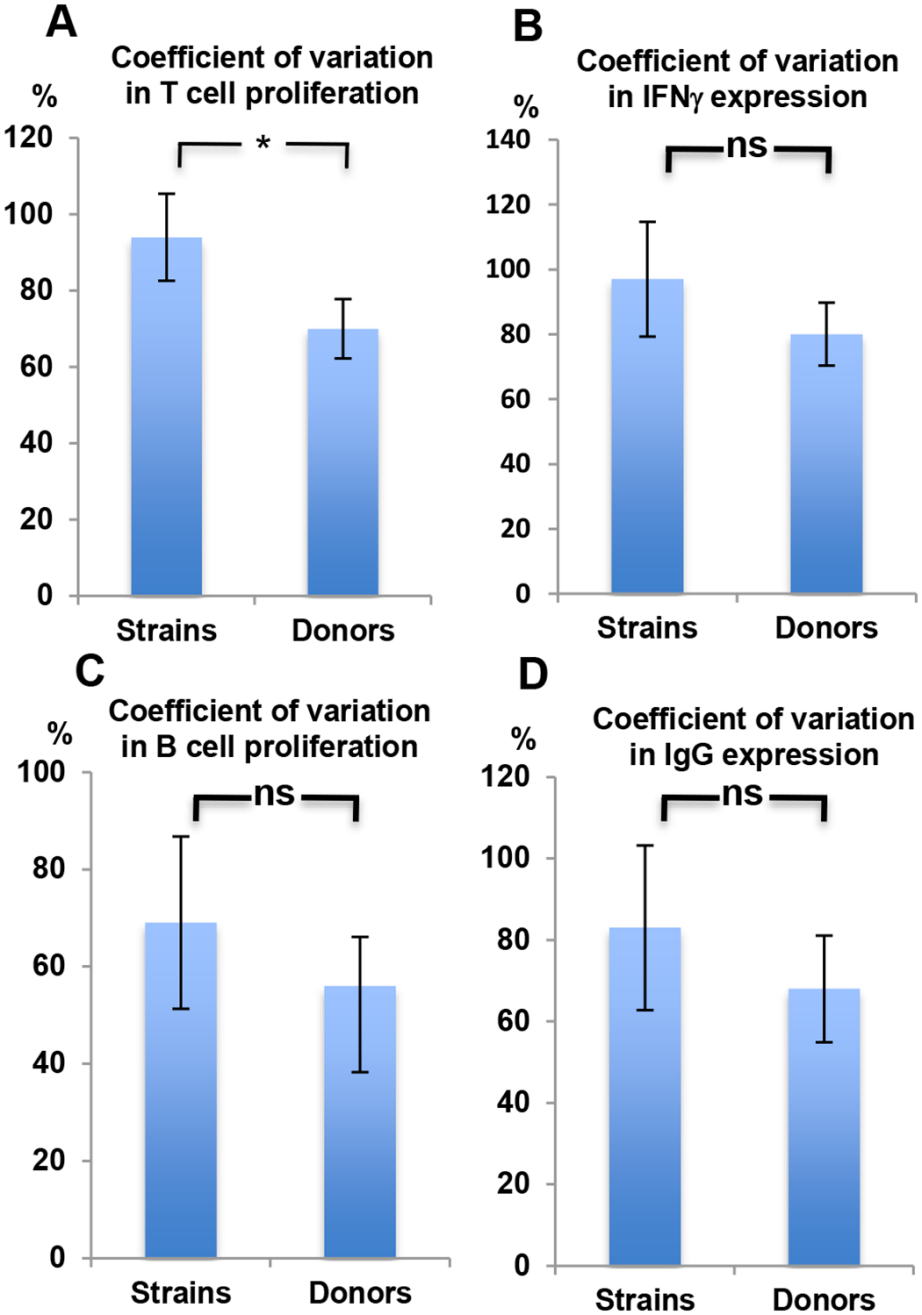
The inter-individual variability of the immune response to a strain vs the intra-individual variability of the immune response to various strains within a species. **A, B, C, and D**. The percentage Coefficient of Variation (CV) in % CD3+CD4+ proliferation (A), %IFNγ expressing CD4+ cells (B), % B cell Proliferation (C), and % IgG expression by proliferating B cells (D), was calculated across bacterial strains vs donors. Expressed is the mean percentage CV±2*SEM.

Can our observed immune response variability to different strains explain part of the inter-individual differences found in response to infection by a specific species, and if so what might this mean clinically? Comparing the Coefficient of Variation of T cell proliferation across donors to that across strains (Fig 4A) and of three other immune adaptive read outs (B cell proliferation, IFNγ, and IgG expressing cells, Fig 4B, 4C and 4D) demonstrates that the contribution of inter-strain variability to the final immune response is at least as large as the contribution of inter-individual variability (see Components of Variance in S8 Table). Moreover, the magnitude of the observed difference in Th17 and Th1 cell induction following stimulation with either Newman or USA600 (Fig 2) is as large as the difference previously described in *S*. *aureus*-infected patients with primary [9, 12] or secondary [13] immune deficiency conditions, respectively, compared to controls. Taken together, the latter two findings further suggest that the clinical heterogeneity observed during infection by a bacterial species may be contributed by the wide variability in the acute adaptive immune response we found in response to individual strains within a species. These results might further fuel the debate between those that emphasize the contribution of human immune variation in determining susceptibility to infections [20] and those who support bacterial genome versatility and diversification as the cause.

In the current study, we evaluated the net effect of the human adaptive immune system 4 days after stimulation, in an attempt to simulate, within the limits of the in-vitro system, the way the adaptive immune system would react to the various strains during an acute infection. The observed wide inter-individual differences in the adaptive responses to an acute infection may represent a combination of the variability in the activation of memory cells (from previous exposure/s to the bacteria), and the activation of new effector cells from naïve cells upon the new encounter with the pathogen. In either case we are evaluating the net adaptive effect of these two populations (naïve and memory) and the combined differential activation by the various strains.

In conclusion, this study demonstrates that the prominent adaptive immune heterogeneity in response to various strains of a bacterial species, as well as the large difference in induction of what is considered to be protective Th1/Th17 immunity [9, 11–13], is contributed by the bacterial accessory genome known to contain a unique combination of virulence determinants and superantigens. Thus, the net immune effect of a strain’s accessory genome “added” to the basic immune response to its "core" (common to all strains of a species) becomes the total unique response to a strain. However, the exact role that the additional response plays towards patient outcome is at this time unknown.

## Materials and methods

### Cells and culture stimulation

Blood from adult healthy donors was obtained at the Rockefeller University hospital. Peripheral blood mononuclear cells (PBMCs) were isolated with Ficoll-Paque Plus (GE Healthcare), after which the cells were stained with Carboxyfluorescein succinimidyl ester (CFSE) and stimulated with heat-killed bacteria in the presence of anti human CD28 (eBioscience, clone 28.2). 4 days later the cells were restimulated for 6 hrs before harvesting and evaluating for proliferating cells.

#### Ethics statement

The study was approved by our IRB committee. All adult subjects provided a written informed consent.

### FACS stain and analysis

The cultured CFSE stained cells were incubated with Brefeldin A (Biolegend) concomitant with their restimulation. 6 hrs later the cells were harvested and stained. For T cell and B cell activation cells were harvested and stained as follow: with Aqua Live/Dead (Life Technologies) anti-human CD3 (PerCP5.5), CD4 (Alex700), CD19 (PE), IgG (BV421). The cell were fixed and permeabilized using BD Cytofix/Cytoperm kit according to the manufacturer’s protocol, after which intra-cellular staining was done with anti-human IFNγ (APC) (all fluorochrome-labeled antibodies from Biolegend). FACS analysis was gated on Live CD3+CD4+CFSE^low^ for proliferating T cells, or Live CD3-CD19+ CFSE^low^ for B cell proliferation. Cells were analyzed by FACS with BD LSRII. The following cell surface markers for T helper (Th) were used CxCR3 (BV421), CCR4 (APC), and CCR6 (PE). LIVE CD3+CD4+ cells were gated on CXCR3+CCR4–CCR6– cells (defined as Th1), CCR6+CCR4+CXCR3– (defined as Th17), CCR6+CXCR3+CCR4– (defined as Th1/Th17), and on CFSE^low^ for proliferation. The following fluorochrome-labeled antibodies were used for intra cellular staining for cytokines or transcription factors: IL-17A (BV421), IFNγ (APC), Tbet (APC), all from Biolegend, IL17F (PE, from eBioscience), RORγt (BD Pharmingen). FACS analysis was done with BD LSRII at our core facility.

### Bacterial strains and preparation

We used 16 strains of *S*. *aureus*. All are clinical isolates (except the lab strain RN4220) that are part of our lab collection obtained from NARSA. The *Streptococcus pyogenes* strains that were used are part of our laboratory’s Lancefield collection. *S*. *aureus* and *S*. *pyogenes* strains were grown over night at 37°C in Tryptic soy (BD) or Todd-Hewitt broth plus 1% Yeast extract, respectively, after which 1:100 dilution of each strain was grown to OD_600_ 0.5 in 20 ml of media. This OD was chosen because the bacteria are at their maximal proliferation stage and before secretion of virulent factors and superantigens [21]. To remove residual virulent factors in the media, the 20 ml of bacterial growth was adjusted to 50 ml with PBS, centrifuged and the bacterial pellet was washed with 50 ml of PBS, then resuspended in PBS to 10^10^ bacteria per ml (based on serial dilution and plating) and were heat killed at 80°C for 1 hr. All strains were maintained in aliquots at -20°C before use.

### Statistical analysis

Statistical analysis was performed with the Prism software (GraphPad). Data represent means ± SEM values, and significance was assessed by nonparametric Mann Whitney test or Friedman test with post-hoc Dunn’s multiple comparison tests. MANOVA test for bivariate analysis was performed with R statistical software. Coefficient of Variation and Components of Variance approach were used to estimate the contribution of donors and strains to the total variability.

## Supporting information

S1 Table Various strains of a species induce a greatly varied acute adaptive immune response. Post-hoc pair-wise comparison of the data found in Fig 1C upper panel. Percent live CD3+CD4+ proliferating cells in response to 16 strains.
(PDF)

S2 Table Post-hoc pairwise comparisons of %IFNγ expressing and proliferating cells by 16 *S*. *aureus* strains. (PDF)

S3 Table Post-hoc pairwise comparisons of percent B cell proliferation in response to 16 *S*. *aureus* strains. (PDF)

S4 Table Post-hoc pairwise comparisons of %IgG expression and B cell proliferating cells in response to 16 *S*. *aureus* strains. (PDF)

S5 Table Complementary to Fig 1H, post-hoc analysis from MANOVA for T cell and B cell proliferation. (PDF)

S6 Table Complementary to Fig 1I, post-hoc analysis from MANOVA for IFNγ and IgG expression. (PDF)

S7 Table Post-hoc analysis from MANOVA for IFNγ and IgG expression. (PDF)

S8 Table The inter-individual variability of the immune response to a strain vs the intra-individual variability of the immune response to various strains within a species. Components of Variance (For relative Contribution of Strains, Donors and Noise to the Total Variability) in R with the use of the package varComp.
(PDF)

S1 Fig Various strains of a species induce a greatly varied acute adaptive immune response. Complementary to Fig 1C lower right panel, %IFNγ expression in live CD3+CD4+ proliferating cells in same 10 donors in response to 16 strains.
(PDF)

S2 Fig Various strains of a species induce a greatly varied acute adaptive immune response. Complementary to Fig 1F left, the percent B cell proliferation in live CD3-CD19+ proliferating cells in 10 donors in response to the 16 strains.
(PDF)

S3 Fig Various strains of a species induce a greatly varied acute adaptive immune response. Complementary to Fig 1F right, %IgG expression and B cell proliferating cells in 10 donors in response to the 16 strains.
(PDF)

S4 Fig Various strains of a species induce a greatly varied acute adaptive immune response. **A**. The MANOVA test for the difference between bivariate means of ranks of IFNγ vs IgG expression among 4 strains, showed significant differences (p<0.00001).
(PDF)

S5 Fig Strains of a species may differ prominently in Th1/Th17 response intensity. Intracellular staining for IFNγ, IL17F expression in proliferating live CD3+CD4+ cells following stimulation of donors with either Newman or USA600 as described in Fig 2.
(PDF)

S6 Fig The effect of different strains on mouse T cell proliferation. Lymph node derived murine PBMC were stained with CFSE, stimulated, cultured, and analyzed by FACS for percent CFSE dilution (%proliferation) in live CD3+CD4+ cells as described with human PBMC in Fig 2A. Expressed are mean +/- SD. * = P<0.05.
(PDF)

S7 Fig Strains of a species may differ prominently in Th1/Th17 response intensity. Intracellular staining for IFNγ, IL17A expression in proliferating live CD3+CD4+ cells following stimulation of donors with *Streptococcus pyogenes* M1, M3, or M6 as described in Fig 2.
(PDF)
