# Supplementary material for: Strains of bacterial species induce a greatly varied acute adaptive immune response: The contribution of the accessory genome
Source: PLoS Pathog. 2018 Jan 11;14(1):e1006726. doi: 10.1371/journal.ppat.1006726 (PMC5764401; doi:10.1371/journal.ppat.1006726)
Supplement: S1 Table — Post-hoc pair-wise comparison of the data found in Fig 1C upper panel. Percent live CD3+CD4+ proliferating cells in response to 16 strains. (PDF) [file ppat.1006726.s001.pdf]

S1 Table

Table 1: post-hoc pairwise comparisons of % CD3+CD4+ T cell proliferation by 16 *S. aureus* strains

	Non Stim	Newman	8325	RN4 220	NRS 111	NRS 112	NRS 113	USA 100	USA 200	USA 300	USA 500	USA 700	USA 800	USA 600	Mu50	VRS2	VRS3A
Non Sti																	
Newman	***																
8325	ns	***															
RN4220	ns	ns	ns														
NRS111	***	ns	***	ns													
NRS112	***	ns	**	ns	ns												
NRS113	ns	ns	ns	ns	ns	ns											
USA100	ns	***	ns	ns	***	ns	ns										
USA200	**	ns	*	ns	ns	ns	ns	ns									
USA300	**	ns	ns	ns	ns	ns	ns	ns	ns								
USA500	***	ns	***	ns	ns	ns	ns	***	ns	ns							
USA700	ns	**	ns	ns	***	ns	ns	ns	ns	ns	**						
USA800	**	ns	*	ns	ns	ns	ns	ns	ns	ns	ns	ns					
USA600	ns	***	ns	ns	***	*	ns	ns	ns	ns	***	ns	ns				
Mu50	***	ns	**	ns	ns	ns	ns	ns	ns	ns	ns	ns	ns	*			
VRS2	ns	*	ns	ns	**	ns	ns	ns	ns	ns	*	ns	ns	ns	ns		
VRS3A	ns	*	ns	ns	**	ns	ns	ns	ns	ns	*	ns	ns	ns	ns	ns	

The post hoc analysis adjusts the p-values for multiple testing. In a selected pairwise comparisons we find that more pairs are significantly different, e.g., comparing RN4220 to Newman using the Mann Whitney test yields a p value= 0.0015 for %CD3+CD4+ T cell proliferation.
