# Supplementary material for: Strains of bacterial species induce a greatly varied acute adaptive immune response: The contribution of the accessory genome
Source: PLoS Pathog. 2018 Jan 11;14(1):e1006726. doi: 10.1371/journal.ppat.1006726 (PMC5764401; doi:10.1371/journal.ppat.1006726)
Supplement: S3 Table — (PDF) [file ppat.1006726.s003.pdf]

S3 Table

Table 3: post-hoc pairwise comparisons of %B cell proliferating cells by 16 *S. aureus* strains

	Non Stim	Newman	8325	RN4 220	NRS 111	NRS 112	NRS 113	USA 100	USA 200	USA 300	USA 500	USA 700	USA 800	USA 600	Mu50	VRS2	VRS3A
Non Sti																	
Newman	***																
8325	ns	***															
RN4220	***	ns	***														
NRS111	***	ns	ns	ns													
NRS112	ns	*	ns	***	ns												
NRS113	ns	**	ns	***	ns	ns											
USA100	***	ns	*	ns	ns	ns	ns										
USA200	ns	ns	ns	ns	ns	ns	ns	ns									
USA300	**	ns	ns	ns	ns	ns	ns	ns	ns								
USA500	***	ns	***	ns	ns	ns	*	ns	ns	ns							
USA700	ns	ns	ns	**	ns	ns	ns	ns	ns	ns	ns						
USA800	**	ns	ns	ns	ns	ns	ns	ns	ns	ns	ns	ns					
USA600	ns	***	ns	***	**	ns	ns	**	ns	*	***	ns	*				
Mu50	ns	**	ns	***	ns	ns	ns	ns	ns	ns	ns	ns	ns	ns			
VRS2	***	ns	*	ns	ns	ns	ns	ns	ns	ns	ns	ns	ns	**	ns		
VRS3A	ns	ns	ns	ns	ns	ns	ns	ns	ns	ns	ns	ns	ns	ns	ns	ns	
