# Supplementary material for: Strains of bacterial species induce a greatly varied acute adaptive immune response: The contribution of the accessory genome
Source: PLoS Pathog. 2018 Jan 11;14(1):e1006726. doi: 10.1371/journal.ppat.1006726 (PMC5764401; doi:10.1371/journal.ppat.1006726)
Supplement: S4 Table — (PDF) [file ppat.1006726.s004.pdf]

S4 Table

Table 4: post-hoc pairwise comparisons of %IgG expressing and proliferating cells by 16 *S. aureus* strains

	Non Stim	Newman	8325	RN4220	NRS 111	NRS112	NRS 113	USA 100	USA 200	USA 300	USA 500	USA 700	USA 800	USA 600	Mu50	VRS2	VRS3A
Non Sti																	
Newman	ns																
8325	ns	ns															
RN4220	***	ns	***														
NRS111	ns	ns	ns	*													
NRS112	ns	ns	ns	***	ns												
NRS113	ns	ns	ns	***	ns	ns											
USA100	***	ns	***	ns	ns	*	ns										
USA200	*	ns	*	ns	ns	ns	ns	ns									
USA300	**	ns	**	ns	ns	ns	ns	ns	ns								
USA500	***	ns	***	ns	ns	***	**	ns	ns	ns							
USA700	ns	ns	ns	***	ns	ns	ns	ns	ns	ns	**						
USA800	ns	ns	ns	*	ns	ns	ns	ns	ns	ns	ns	ns					
USA600	ns	ns	ns	***	ns	ns	ns	***	*	**	***	ns	ns				
Mu50	ns	ns	ns	***	ns	ns	ns	ns	ns	ns	**	ns	ns	ns			
VRS2	***	ns	***	ns	ns	*	ns	ns	ns	ns	ns	ns	ns	***	ns		
VRS3A	*	ns	*	ns	ns	ns	ns	ns	ns	ns	ns	ns	ns	*	ns	ns	
