# Supplementary material for: Strains of bacterial species induce a greatly varied acute adaptive immune response: The contribution of the accessory genome
Source: PLoS Pathog. 2018 Jan 11;14(1):e1006726. doi: 10.1371/journal.ppat.1006726 (PMC5764401; doi:10.1371/journal.ppat.1006726)
Supplement: S5 Table — (PDF) [file ppat.1006726.s005.pdf]

S5 Table

Table 5:
Post-hoc analysis from MANOVA for T cell and B cell proliferation

contrast	estimate	SE	df	t. ratio	p.value
Mu50 - Newman	-6.125	1.025474	36	-5.973	<.0001
Mu50 - USA100	-2.625	1.025474	36	0.634	0.5302
Mu50 - USA600	5.675	1.025474	36	5.534	<.0001
Newman - USA100	6.775	1.025474	36	6.607	<.0001
Newman - USA600	11.800	1.025474	36	11.507	<.0001
USA100 - USA600	8.075	1.025474	36	4.900	<.0001
