# Supplementary material for: Strains of bacterial species induce a greatly varied acute adaptive immune response: The contribution of the accessory genome
Source: PLoS Pathog. 2018 Jan 11;14(1):e1006726. doi: 10.1371/journal.ppat.1006726 (PMC5764401; doi:10.1371/journal.ppat.1006726)
Supplement: S6 Table — (PDF) [file ppat.1006726.s006.pdf]

S6 Table

Table 6:
Post-hoc analysis from MANOVA for IFNg and IgG expression

contrast	estimate	SE	df	t. ratio	p.value
Mu50 - Newman	-3.275	0.9896338	36	-3.309	0.0032
Mu50 - USA100	-2.625	0.9896338	36	-2.652	0.0142
Mu50 - USA600	5.450	0.9896338	36	5.507	<.0001
Newman - USA100	0.650	0.9896338	36	0.657	0.5155
Newman - USA600	8.725	0.9896338	36	8.816	<.0001
USA100 - USA600	8.075	0.9896338	36	8.160	<.0001
