# Supplementary material for: Strains of bacterial species induce a greatly varied acute adaptive immune response: The contribution of the accessory genome
Source: PLoS Pathog. 2018 Jan 11;14(1):e1006726. doi: 10.1371/journal.ppat.1006726 (PMC5764401; doi:10.1371/journal.ppat.1006726)
Supplement: S7 Table — (PDF) [file ppat.1006726.s007.pdf]

S7 Table

Table 7:
Post-hoc analysis from MANOVA for IFNg and IgG expression

contrast	estimate	SE	df	t. ratio	p.value
Mu50 - USA500	-6.375	0.9799731	36	-6.505	<.0001
Mu50 - USA100	-2.625	0.9799731	36	-2.679	0.0111
Mu50 - USA600	5.450	0.9799731	36	5.561	<.0001
USA500 - USA100	3.750	0.9799731	36	3.827	0.0006
USA500 - USA600	11.825	0.9799731	36	12.067	<.0001
USA100 - USA600	8.075	0.9799731	36	8.240	<.0001
