# Supplementary material for: Strains of bacterial species induce a greatly varied acute adaptive immune response: The contribution of the accessory genome
Source: PLoS Pathog. 2018 Jan 11;14(1):e1006726. doi: 10.1371/journal.ppat.1006726 (PMC5764401; doi:10.1371/journal.ppat.1006726)
Supplement: S8 Table — Components of Variance (For relative Contribution of Strains, Donors and Noise to the Total Variability) in R with the use of the package varComp. (PDF) [file ppat.1006726.s008.pdf]

**S8 Table**

**Components of Variance* (For relative Contribution of Strains, Donors and Noise to the Total Variability)**

| | strains | donors | noise | p-value |
|---|---|---|---|---|
| %T cell prolife | 0.47344415 | 0.2921156 | 0.23444025 | 0.07436448 |
| %IFNg | 0.33923254 | 0.24116854 | 0.41959892 | 0.40348084 |
| %B cell prolife | 0.47314102 | 0.34925579 | 0.17760319 | 0.20246055 |
| %IgG | 0.5642693 | 0.26679564 | 0.16893506 | 0.0017798 |

*The components of variance were estimated in R with the use of the package varComp, after fitting a linear mixed-effects model that used donors and strains as additive random effects. The table shows the percentage of the variance estimated for each component, i.e. donors and strains, in the model and the remaining variance of residuals (random noise).
