# Supplementary figures and images for: Strains of bacterial species induce a greatly varied acute adaptive immune response: The contribution of the accessory genome
Source: PLoS Pathog. 2018 Jan 11;14(1):e1006726. doi: 10.1371/journal.ppat.1006726 (PMC5764401; doi:10.1371/journal.ppat.1006726)

**S1 Fig**

**%IFN $\gamma$  expressing and proliferating CD3+CD4+ cells in response to 16 strains in 10 donors.**

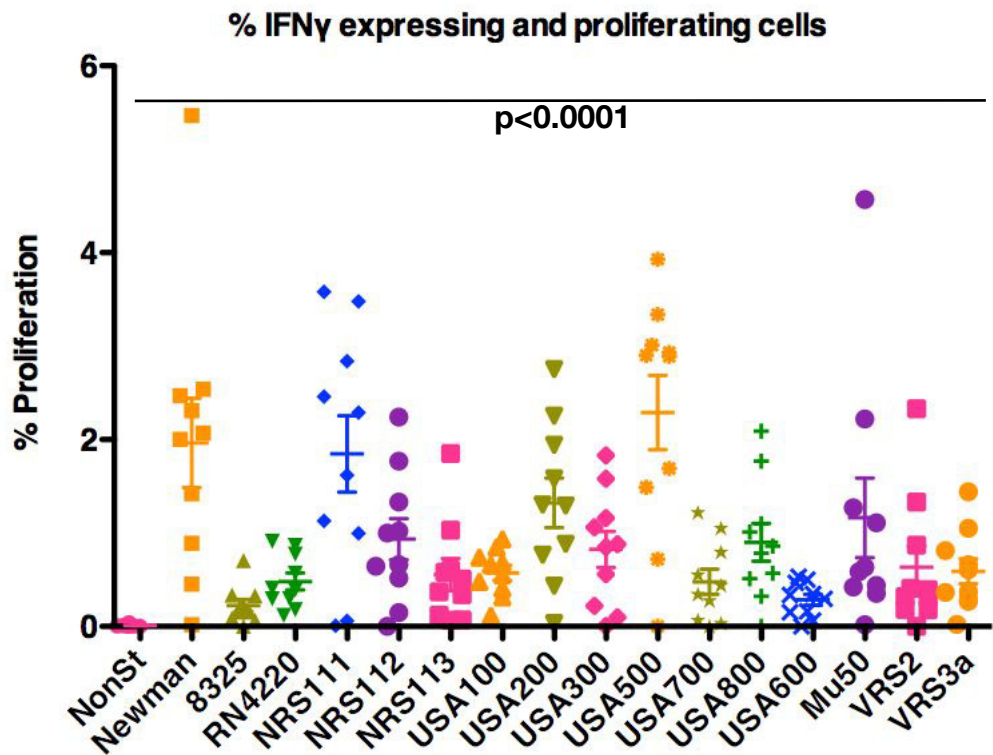

Supplement: S1 Fig — Complementary to Fig 1C lower right panel, %IFNγ expression in live CD3+CD4+ proliferating cells in same 10 donors in response to 16 strains. (PDF) [file ppat.1006726.s009.pdf]

S2 Fig

% B cell proliferation in 10 donors in response to 16 *S. aureus* strains

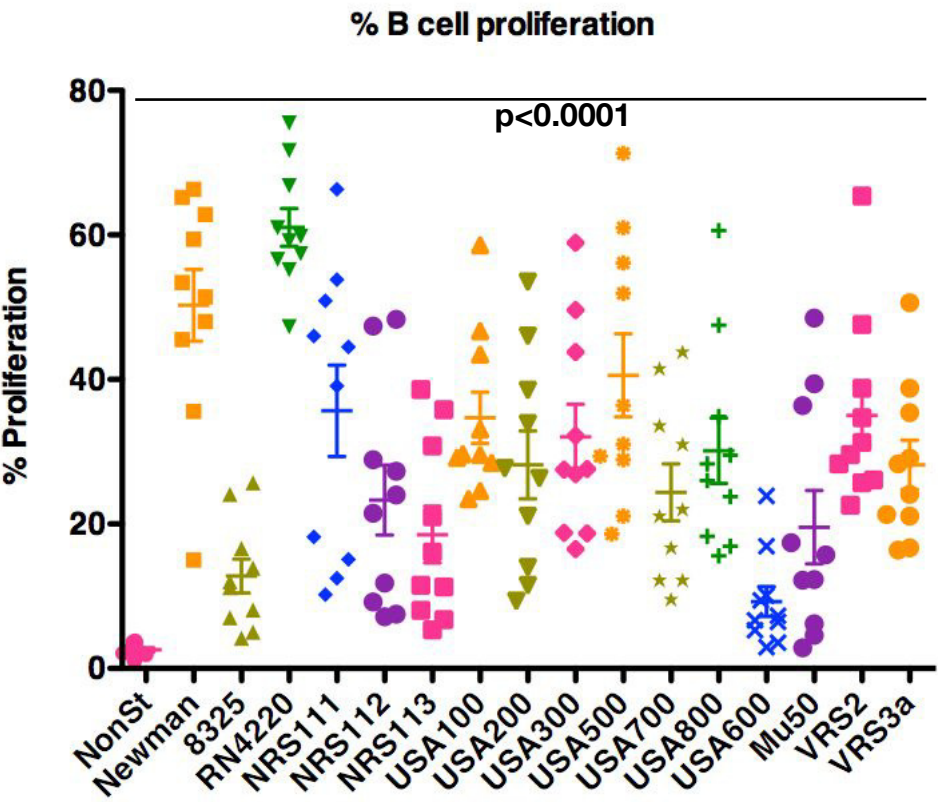

Supplement: S2 Fig — Complementary to Fig 1F left, the percent B cell proliferation in live CD3-CD19+ proliferating cells in 10 donors in response to the 16 strains. (PDF) [file ppat.1006726.s010.pdf]

S3 Fig

% IgG expressing and B cell proliferating cells in 10 donors in response to 16 *S. aureus* strains

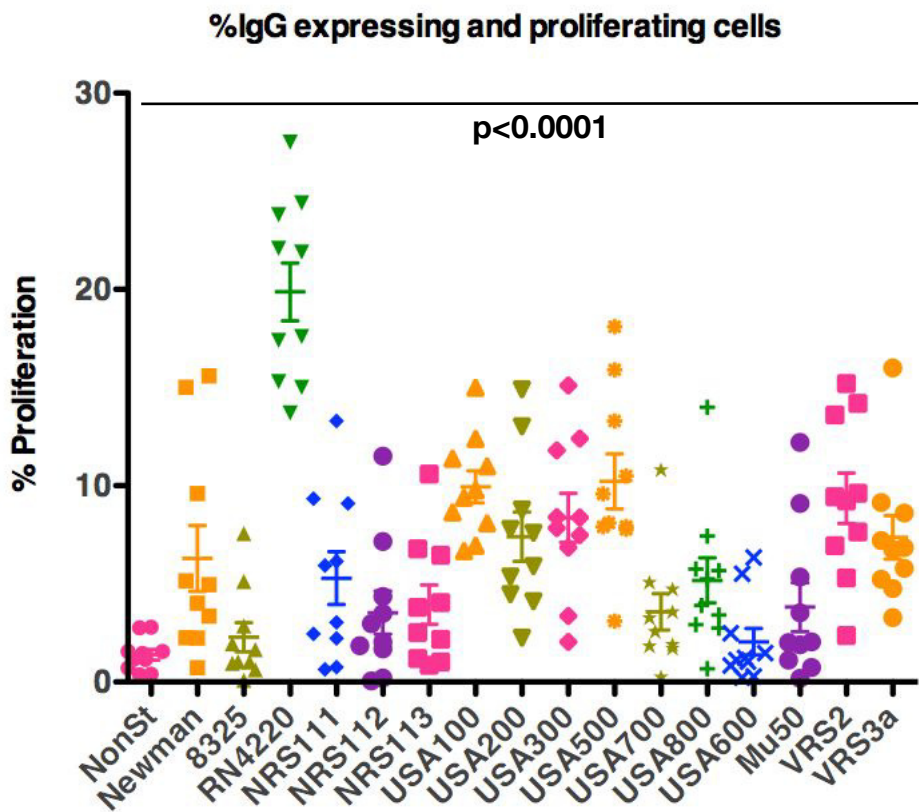

Supplement: S3 Fig — Complementary to Fig 1F right, %IgG expression and B cell proliferating cells in 10 donors in response to the 16 strains. (PDF) [file ppat.1006726.s011.pdf]

S4 Fig

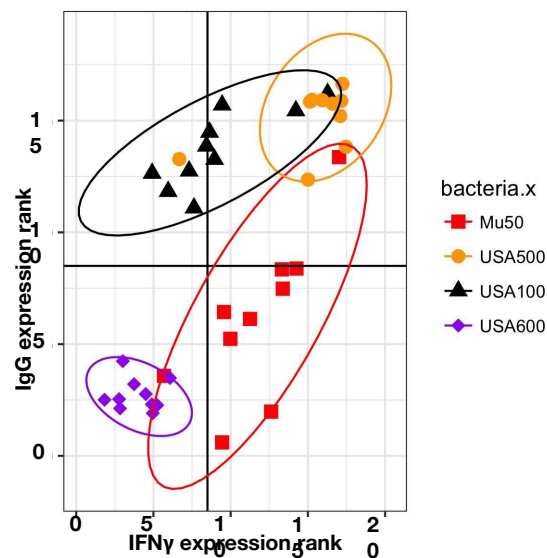

Supplement: S4 Fig — A. The MANOVA test for the difference between bivariate means of ranks of IFNγ vs IgG expression among 4 strains, showed significant differences (p<0.00001). (PDF) [file ppat.1006726.s012.pdf]

S5 Fig

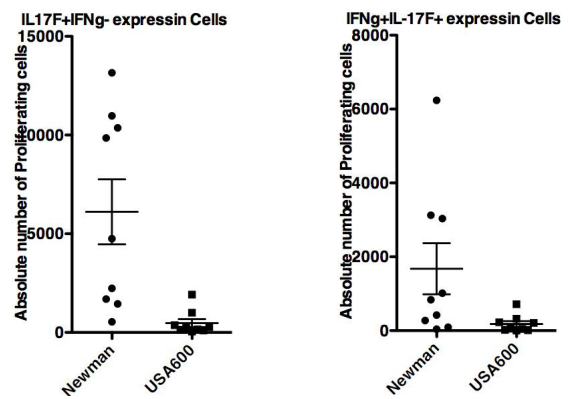

Supplement: S5 Fig — Intracellular staining for IFNγ, IL17F expression in proliferating live CD3+CD4+ cells following stimulation of donors with either Newman or USA600 as described in Fig 2. (PDF) [file ppat.1006726.s013.pdf]

S6 Fig

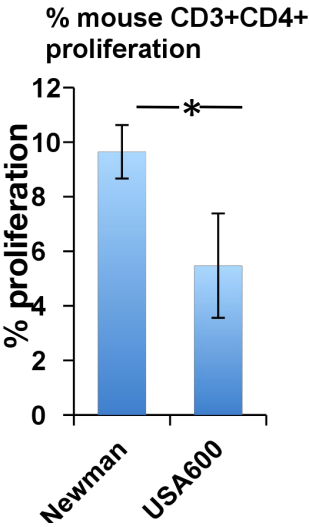

Supplement: S6 Fig — Lymph node derived murine PBMC were stained with CFSE, stimulated, cultured, and analyzed by FACS for percent CFSE dilution (%proliferation) in live CD3+CD4+ cells as described with human PBMC in Fig 2A. Expressed are mean +/- SD. * = P<0.05. (PDF) [file ppat.1006726.s014.pdf]

S7 Fig

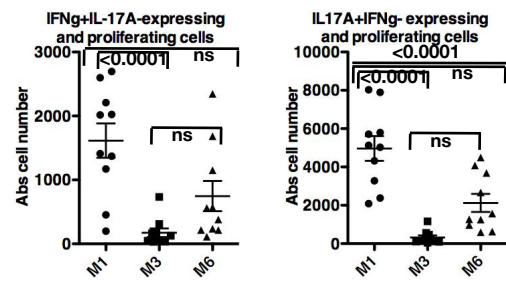

Supplement: S7 Fig — Intracellular staining for IFNγ, IL17A expression in proliferating live CD3+CD4+ cells following stimulation of donors with Streptococcus pyogenes M1, M3, or M6 as described in Fig 2. (PDF) [file ppat.1006726.s015.pdf]
